# The Potential Role of SARS-COV-2 in the Pathogenesis of Parkinson's Disease

**DOI:** 10.3389/fneur.2020.01044

**Published:** 2020-09-17

**Authors:** Pedro Chaná-Cuevas, Philippe Salles-Gándara, Alejandro Rojas-Fernandez, Constanza Salinas-Rebolledo, Anna Milán-Solé

**Affiliations:** ^1^Movement Disorders Center, CETRAM, Santiago, Chile; ^2^Faculty of Medical Science, University of Santiago of Chile, Santiago, Chile; ^3^Institute of Medicine & Center for Interdisciplinary Studies on the Nervous System (CISNe), Universidad Austral de Chile, Valdivia, Chile; ^4^Liga Chilena Contra la Epilepsia (LICHE), Santiago, Chile

**Keywords:** coronavirus, SARS virus, nervous system diseases, movement disorders, Parkinson disease, alpha-synuclein, neurodegenerative diseases, pandemics

## Abstract

Considering their current burden and epidemiological projections, nowadays Parkinson's disease and the COVID-19 pandemic are two key health problems. There is evidence of the pathogenic role of neurotropic viruses in neurodegenerative diseases and coronaviruses are neurotropic, with some of them selectively targeting the basal ganglia. Moreover, some authors demonstrated the longevity of these viruses in the affected cells of the nervous system for long periods. Coronavirus was detected in brain autopsies and SARS-CoV-2 has been isolated from the CSF of affected patients. The marked inflammatory response in some particular patients with COVID-19 with a consequent increase of pro-inflammatory cytokines is considered a prognostic factor. Immunologic changes are observed in patients with Parkinson's disease, possibly having a role in its pathogenesis. A dynamic pro-inflammatory state accompanies α-synuclein accumulation and the development and progression of neurodegeneration. Also, some viral infectious diseases might have a role as triggers, generating a cross autoimmune reaction against α-synuclein. In the past Coronaviruses have been related to Parkinson's disease, however, until now the causal role of these viruses is unknown. In this paper, our focus is to assess the potential relationship between SARS-CoV-2 infection and Parkinson's disease.

## Introduction

Parkinson's disease (PD) is the second most common and the most rapidly growing neurodegenerative disorder (1). Its pathological hallmarks are loss of dopaminergic neurons in the substantia nigra (SN) pars compacta and accumulation of misfolded α-synuclein, which is found in intracytoplasmic inclusions called Lewy bodies (2).

The current global burden of PD is about 6.2 million cases (3), and it is expected that more than 12 million people worldwide will be affected by the year 2040 (1, 4). This exponential growth worldwide may be attributed to several factors (5), including infectious diseases. A recent analysis suggests that viral and bacterial infections might increase the risk of developing PD (6).

The hypothesis of a viral trigger associated with the pathogenesis of PD emerged more than 100 years ago, due to the relation of lethargic encephalitis (Von Economo disease) and post-encephalitic parkinsonism that occurred after the 1918 type A H1N1 influenza pandemic (7). Until now, influenza remains the main basis of the viral hypothesis, supported by its neurotrophic properties, with preferential targets in the SN and ventral tegmental area (8); and the finding of MxA protein in Lewy bodies, which is implicated in the defense against influenza (9). In the last decades, additional viruses have been associated with both acute and chronic parkinsonism, including Epstein Barr virus, Coxsackie, Japanese encephalitis B, western equine encephalitis, West Nile virus, herpes viruses, and HIV (8, 10, 11).

The contemporary pandemic, starting from December 2019 to date, of severe acute respiratory syndrome coronavirus 2 (SARS-CoV-2) which is responsible for coronavirus disease (COVID-19), is now a worldwide health concern (10). With more than 16 million COVID-19 cases globally (July 28, 2020)^1^, our attention is now set on the hypothetical relations of this new coronavirus infection on PD pathogenesis, its potential as a trigger for the neurodegenerative process, and its consequent impact on the epidemiology of PD. One of the elements that set off this alarm was the report of anosmia in patients infected by SARS-CoV-2, but also the neuroinvasive potential of coronaviruses (CoVs) and a noticeable inflammatory reaction in severe COVID-19 cases. As we know, immune activation in the peripheral and central nervous system (CNS) is a common finding in cases of PD (12, 13). Moreover, inflammation can trigger α-synuclein misfolding, aggregation, and propagation through the CNS (14–16). α-synuclein aggregation may activate microglia, favoring the pro-inflammatory response and cell damage signals, which ultimately leads to neuronal death. In this hypothetical scenario, older adults may represent a susceptible group to the development of neurodegenerative disorders, as aging might be associated with low-grade and chronic inflammation (“inflammaging”) (16), and the inability to control inflammation (17).

Exploring the potential relationship of SARS-CoV-2 and PD is essential because of the epidemiological implications and the understanding of physio pathological aspects of both disorders. Our paper attempts to elucidate some of those hypothetical links and its possible consequences.

### The Neuroinvasive Potential of SARS-CoV-2

Most CoVs share a similar viral structure, infection route, and pathogenic mechanism. The penetration of the virus in host cells is mediated by the angiotensin-converting enzyme 2 (ACE2), and dipeptidyl peptidase 4 (DPP4) (18). In addition to the severe acute respiratory syndrome, human CoV infections may manifest severe neurological complications including seizures, refractory status epilepticus, encephalitis, acute disseminated encephalomyelitis, cerebellitis, Guillan-Barré syndrome, leukoencephalopathy, and critical illness neuromyopathy (19).

Not unexpectedly, evidence shows that neuroinvasion and neurotropism is one common feature of CoVs. Such neuroinvasive propensity has been documented for most βCoVs, including SARS-CoV, MERS-CoV, HCoV-229E, HCoV-OC43, mouse hepatitis virus (MHV), and porcine hemagglutinating encephalomyelitis coronavirus (HEV) (18).

As an example of CNS invasion, following intranasal inoculation of susceptible mice, HCoV-OC43 infects the olfactory bulb and disseminates to the hippocampus and cortex, from which it appears to spread by the trans-neuronal route to the brainstem (20). Meanwhile, Fishman et al., observed a strong tropism for the basal ganglia in the region of the subthalamic nucleus and SN in MHV-A59-infected C57BL/6 mice, with fewer signs of infection in other brain regions (21). Alongside, Arbor et al. demonstrated the potential chronic persistence of HCoV-229E and HCoV-OC43 infection in human neuronal cell lines, specially oligodendrocytes, and possibly neurons (22). Further, HCoV-OC43 RNA can be detected for over a year in the CNS of infected mice that survived the acute encephalitis (23).

CNS invasion was also demonstrated in humans. Gu et al. (24) reported a postmortem study of patients who died 14–62 days after the onset of SARS symptoms. Brain edema and scattered red degeneration of neurons affected the brains in 6 of 8 confirmed cases. Moreover, the presence of virus confined to the cytoplasm of numerous neurons in the hypothalamus and cortex was confirmed by light microscopy, electron microscopy, and real-time PCR (24). The average time from symptom onset to hospital admission is 7 days, while the average time of admission to the intensive care unit is 8 days (25). This latency may represent the “window of time” for the virus to enter the CNS (26).

Similar to other CoVs, SARS-CoV-2 infects cells through the interaction between its spike protein (S) and ACE2. For this interaction, protein S must be cleaved by Transmembrane Serine Protease (TMPRSS2) (27, 28). Cells expressing both ACE2 and TMPRSS2 are more susceptible to SARS-CoV-2 infection (29). Recently, Chen et al. investigated ACE2 expression by analyzing data from brain transcriptome databases. The SARS-CoV-2 receptor was highly expressed in the SN and brain ventricles, and distributed in excitatory as well as inhibitory neurons, but also astrocytes and oligodendrocytes (30).

Although there is no evidence of strong co-expression of ACE2^+^/TMPRSS2^+^ in the brain (29), Brann et al. showed that non-neuronal cells of the sensory olfactory epithelium (sustentacular cells, horizontal basal cells, microvillar cells, and Bowman's gland cells) express both ACE2 and TMPRSS2. Human sustentacular cells express both genes at levels comparable to those observed in lung cells. Thus, these cells could be the first to be infected with SARS-CoV-2 (27). These non-neuronal cells support mature olfactory sensory neurons (OSNs) in the sensory epithelium. Supporting cells infected by SARS-CoV-2 could eventually spread the virus to OSNs through axonal transport (31), later invading neurons within the olfactory bulb and then to the CNS causing inflammation (32). The mechanism of viral penetration through the olfactory bulb into the brain has been previously proposed to play a role in neurodegenerative diseases, acting as a trigger for the spread of pathologically aggregated proteins in a prion-like manner (33).

In a retrospective analysis of patients hospitalized with COVID-19 in Wuhan, China, the authors found that of 214 cases, 78 had neurologic manifestations, including impaired consciousness and cerebrovascular diseases, with a higher prevalence in more severe cases (34, 35). Also, anosmia and dysgeusia are commonly reported in COVID-19 patients (36). Recently, cerebrospinal fluid (CSF) samples from patients with COVID-19 presenting meningitis and encephalitis were positive for SARS-CoV-2 (37). The nasopharyngeal sample from one patient with meningitis was negative in the RT-PCR test for SARS-CoV-2, but a CSF sample resulted positive for the virus (38). These findings support the hypothesis that SARS-CoV-2 like other CoVs has the potential to infect brain cells (39).

Interestingly, SARS- CoV-2 RNA was also detected in the feces in ~50% of patients with COVID-19. Moreover, there is evidence of intestinal inflammation in these patients (40). These findings recall the model of gut-driven inflammation in PD pathogenesis. In this model an initial infection, which directly or indirectly affects the GI system, triggers an inflammatory response, increasing the levels of α-synuclein in the gut and brain, which would initiate its aggregation (41).

In our opinion, these findings support the neuroinvasive potential of SARS-CoV-2 similar to other coronaviruses. The ability of CoVs to remain for long periods in the CNS could perpetuate the central inflammatory response and the risk of neurodegeneration. The more or less selective invasion of the SN and basal ganglia could be partially explained by a high local expression of ACE2, resembling pathologically affected areas in PD. Finally, invasion through the olfactory bulb and evidence of intestinal inflammation in COVID-19 patients reflects on the Braak's hypothesis, and the model of gut-origin of PD.

### SARS-CoV-2 and the Inflammatory Response

A fast and synchronized innate immune response is the first line of defense against viral infections. On the contrary, dysregulated and exaggerated immune reactions may cause immune damage to the human body (42).

For example, in the SARS-CoV epidemic, cerebral involvement was related to the exaggerated viral immune response. A study reported a high ratio of monokine induced by IFN-γ (Mig), and IFN-γ inducible protein 10 (IP 10) in the blood of patients with SARS, and an increase in Mig but not IP-10 in brain tissue, which in turn seems to attract CD68+ macrophages and CD3+ lymphocytes to the sites of virus infection; contributing to brain damage (43). This effect is mediated by NF-κB. Its pharmacological inhibition markedly decreased Mig in the affected organs (44). Drugs like Bortezomib and other proteasome inhibitors possess this inhibitory potential and could eventually regulate the inflammatory response (45).

In COVID-19, high levels of IL-1B, IFN-γ, IP-10, and monocyte chemoattractant protein 1 (MCP-1) have been detected. These cytokines may activate the T-helper type 1 (Th1) cell response, a key event in the activation of specific immunity. Nevertheless, contrasting to SARS cases, patients with COVID-19 also have elevated levels of Th2 cell-secreted cytokines (such as IL-4 and IL-10), which inhibit the inflammatory response (44, 45).

Current evidence indicates that some of the COVID-19 patients present characteristics similar to secondary adult hemophagocytic syndrome, including cytopenia (46, 47) and cytokine storm syndrome (48, 49). This inflammatory cytokine storm is closely associated with the development of acute respiratory distress syndrome and extrapulmonary multiple-organ failure. Significantly high blood levels of cytokines and chemokines were detected in patients with severe cases of COVID-19 admitted to the intensive care unit, including IL2, IL7, IL10, GCSF, IP10, MCP1, MIP1α, and TNFα which are believed to promote disease severity (47). The cytokine serum levels, specially IL-2R and IL-6 in patients with COVID-19, positively correlate with mortality rate (46). Ruan et al. conducted a retrospective study in 150 laboratory-confirmed Chinese patients with SARS-CoV-2. They observed elevated IL-6 levels in non-survivors compared to those with mild infection (50). Moreover, a case of COVID-19-associated acute necrotizing hemorrhagic encephalopathy (ANE) was recently reported (51). This type of encephalopathy is a rare complication in other viral infections, associated with intracranial cytokine storm and a breakdown of the blood-brain barrier, rather than a direct viral invasion (52). Previous studies showed that an exaggerated and dysregulated cytokine response leads to neuronal death (53).

From our viewpoint, the importance of these findings lies in previous evidence indicating that exaggerated or prolonged systemic inflammation alone is sufficient to pathologically modify α-synuclein in the CNS. Moreover, peripheral inflammation may also increase α-synuclein uptake from the circulation into the brain by promoting disruption of the blood-brain barrier (54); the increased permeability of the blood-brain barrier facilitates lymphocyte infiltration into the CNS, and microglial activation, which is a hallmark in neurodegenerative diseases (55). Also, peripheral inflammation may exacerbate the central brain's ongoing damage in several neurodegenerative diseases (56).

### Inmunologic Variations in Parkinson's Disease

Certainly, immune activation is an important piece in the puzzle of PD physiopathology. CNS immune changes are characterized mainly by reactive microgliosis and high concentrations of pro-inflammatory cytokines. Similarly, an imbalance in lymphocyte populations favors a TH1-type peripheral system immune response.

As we pointed out, brain autopsies of PD cases show microglial and oligodendroglial activation and upregulation of major histocompatibility class II (MHCII). Activated microglia in the putamen expressed TNF-alpha and IL-6, remarkably, these inflammatory cytokines may also have a neurotrophic role. The expression of these factors is concomitant with α-synuclein accumulation and loss of dopaminergic cells in the SN (57). Meanwhile, Mogi et al. reported higher concentrations of IL-1ß, IL-6, epidermal growth factor (EGF), and transforming growth factor-alpha (TGF-alpha) in striatal regions in the brain of PD cases compared with controls. IL-1ß, an immune response-generated cytokine, stimulates astrocyte proliferation, while IL-6 is a B-cell stimulating factor. At the same time, astrocytes as well as microglial cells secrete IL-1ß and IL-6 (58).

Baba et al. analyzed T-lymphocyte populations in patients with PD. They found a characteristic predominant expression of CD8^+^ T cells, depletion of CD4^+^ CD25^+^^highcells^, and a shift to a TH1-type peripheral immune system (13). Also, Stevens et al., found a 15–25% reduction in TCRαβ+, CD4+ (T helpers), and CD19+ (B) cells compared to controls (59).

A meta-analysis from 25 studies involving 1,547 patients with PD and 1,107 controls, was consistent with elevated peripheral concentrations of several inflammatory cytokines, including, IL-6, TNF, IL-1β, IL-2, IL-10, CRP, and RANTES in patients with PD (60). These changes might be associated with the inflammatory process in the brain.

Additionally, various genetic loci were identified in genome-wide association studies as risk factors for PD, some within the HLA region, coding for immune genes including MHCII (61, 62), particularly the rs3129882 single nucleotide polymorphism (SNP). The GG homozygosity of this SNP is associated with increased baseline and inducible MHC-II expression in APCs, favoring a more pro-inflammatory CD4+ T cell response (63).

It is not clear if these changes in the immune system are the cause or consequence of an initial trigger for the neurodegenerative process: based on what was previously stated, the inflammatory insult associated with SARS-CoV-2 infection could be a predisposing factor, particularly in susceptible individuals. Moreover, immunologic variations in PD patients may affect their outcome after SARS-CoV-2 infection.

### The Neuroinflamatory Response and Synucleinopathy

The normal function of α-synuclein is partially understood. Part of its role involves the recycling of synaptic vesicles and synaptic transmission, as it is abundant in synaptic clefts (64, 65). Consequently, the loss of its normal neuronal function could play a central role in PD pathophysiology.

Current evidence suggests different pathogens as triggers of a cerebral chronic neuroinflammatory response (66, 67); α-synuclein is involved in important aspects of immune activation, specifically with the innate immune response. It may have a regulatory role in the immune response of peripheric and central neurons (68–70) and could be involved in the canonic activation of inflammatory pathways (inflammation), as well as the chronic immune response and neurotoxicity (neurodegeneration) (71). This occurs due to the overexpression of Toll-like receptors (TLR) and Nuclear Factor (NF-κB), activating, in turn, the cytokine response cascade. The presence of extracellular α-synuclein is a marker of molecular damage (72).

**Figure 1:**
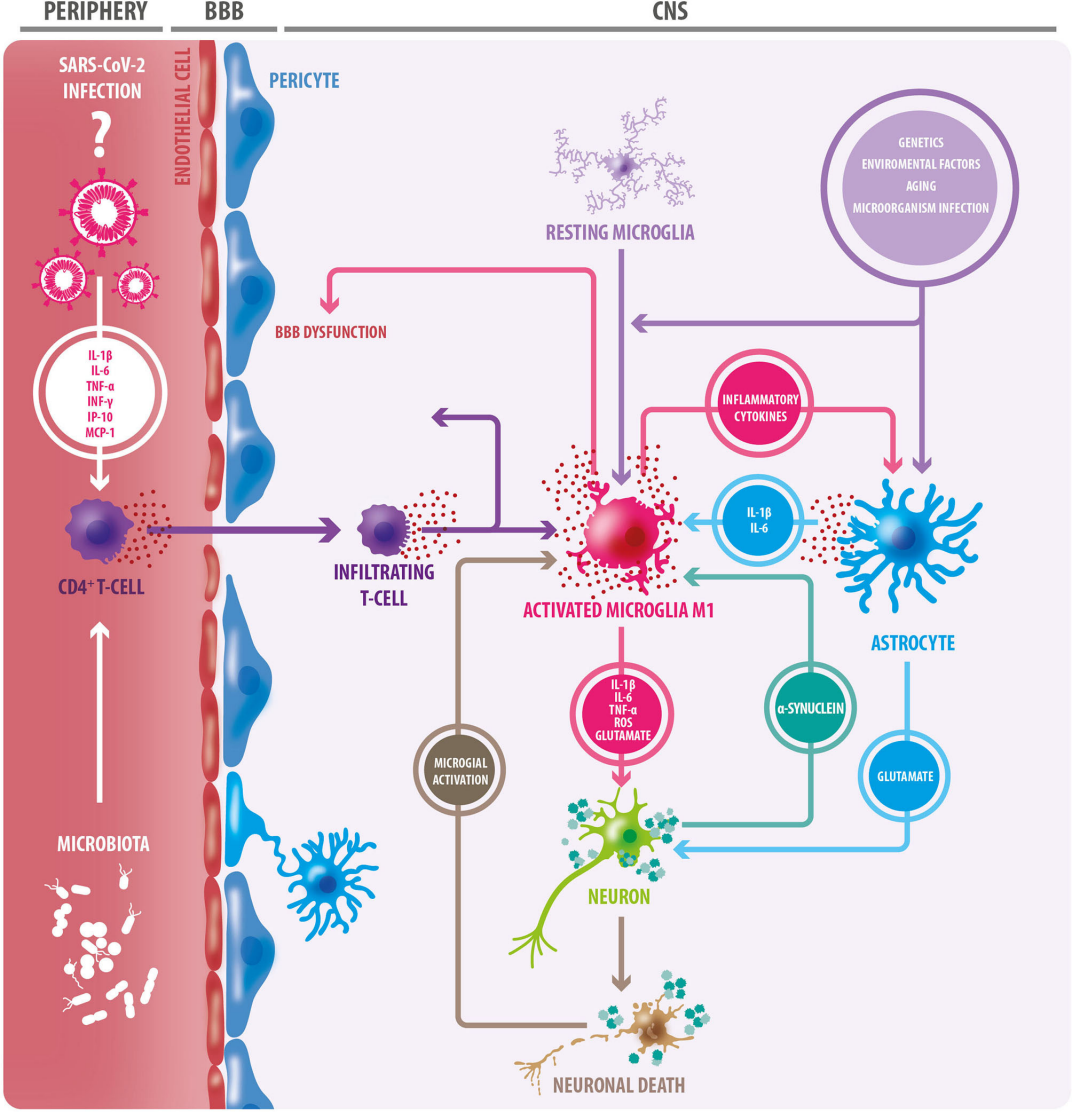
Triggering factors of the neuroinflammatory process. Aging, in addition to genetic and environmental factors, and infections of certain microorganisms, can trigger a neuroinflammatory response through microglial and oligodendroglial activation. Activated microglia adopt an M1 inflammatory phenotype, secreting proinflammatory cytokines, reactive oxygen species (ROS), and glutamate; factors that cause neuronal damage. In this context, astrocytes become reactive, and like microglia, they secrete proinflammatory cytokines. Many of these cytokines act on microglial cells, exacerbating microglial activation, and favoring neuronal damage. The release of TNF-alpha by microglia induces increased glutamate release by astrocytes: a detrimental event for neurons. In this context, degenerating and/or dead neurons are observed, which in turn trigger microglial activation. Protein accumulation (e.g., alpha-synuclein) is another triggering factor for microglial activation. Microglia degrades and presents components of dead cells and protein aggregates to CD4+ T lymphocytes. This, in conjunction with the release of cytokines, results in the infiltration of CD4 + T cells, which release more proinflammatory cytokines, leading to greater neurodegeneration. As a consequence of this neuroinflammation, the blood-brain barrier (BBB) becomes dysfunctional, leading to the entry of peripheral immune cells. In the periphery, gut microbiota can trigger inflammation mediated by innate immune cells. The SARS-CoV-2 virus generates a “cytokine storm” at the peripheral level, therefore, it could have a similar effect. Inflammatory cytokines from peripheral blood circulation could also contribute to BBB permeabilization.

There is also evidence to suggest that α-synuclein plays a role in mechanisms of infection responses, with an increased expression of α-synuclein in viral processes such as in Nile Virus encephalitis (73), and worse disease prognosis in α-synuclein knockout mice (74). During viral infections, α-synuclein increases, acting as an inhibitor of viral growth in neurons in the CNS by acting as a restricting factor of viral RNA (75). TLR are a group of transmembrane glycoproteins implicated in pathogen recognition and immune response, which are regulated by α-synuclein, as well as other immune mechanisms (76). α-synuclein can function as an antigen associated with cellular damage and can be recognized by TLR 1 to 4, 7, and 8. The activation and potentiation of inflammatory responses are related to TLR 2 and 3, with a magnifying effect of Interferon γ (IFN-γ) (72, 73, 77), suggesting that the activation of the immune response, could lead to a chronic inflammatory process (76, 77). Finally, one hypothesis debates that infectious processes may generate an autoimmune response against α-synuclein (78) (Figure 1).

### Evidence of the Relation of SARS-CoV-2 and Parkinson's Disease

As we mentioned previously, proinflammatory events such as viral infections are proposed as predisposing factors for individuals to develop PD and long-term neuronal loss (79). Special consideration regarding SARS-CoV-2 is its capacity to induce a marked systemic pro-inflammatory response. A prospective case-control study showed that men with higher plasma IL-6 concentrations had an increased risk of developing PD (80). As stated above, this interleukin is highly elevated in COVID-19. Therefore, it is necessary to determine the persistence of high IL-6 levels in recovered COVID-19 patients.

As we know, SARS-CoV-2 infects cells through ACE2 and TMPRSS2. Interestingly, Li et al. found that TMPRSS2 is up-regulated in rats treated with 6-hydroxydopamine (6-OHDA), a widely used tool to model PD, compared to control rats. This study showed that genes codifying for this protein are differentially regulated and may play an important role in the development of the disease (81). Surprisingly, in the past CoVs were related to PD patients. Specifically, intrathecal antibodies for CoVs types MHV-JHM and MHV-A59 are elevated in PD patients compared to individuals with other neurological diseases (82). However, the causal role of these viruses in PD is still unknown.

Although we are waiting for a longer follow-up period of recovered COVID-19 patients, some features in the acute phase of the disease are very striking. For example, anosmia and gastrointestinal symptoms are common early findings (34, 83); and a high prevalence of impaired consciousness was observed in more severe cases (34). It was thought that its neurotropic affinity could be related to its ability to produce respiratory symptoms, with over 89% of patients in the intensive care units unable to generate spontaneous ventilation, putatively due to central dysfunction (18). Hyposmia and gastrointestinal manifestations are also common non-motor symptoms in PD during the prodromal phase, a period during which neurodegeneration has begun (84–86). According to Braak's hypothesis, these symptoms represent the first stage of PD which involves the deposition of α-synuclein in the anterior olfactory nucleus and dorsal motor nucleus of the vagus (87). We could then presuppose an overlap in the anatomical distribution of the initial pathological process of both diseases.

## Conclusion

The ongoing COVID-19 pandemic is expected to affect a large amount of the world‘s population. Although we have more clarity about its acute behavior, the chronic effects of this virus are yet to be seen, since a comprehensive understanding of SARS-CoV-2 is still lacking. The systemic inflammatory response induced by SARS-CoV-2 seems enough to set off the alarms on its potential relation with neuroinflammation, but also cumulative evidence supports its neurotropic capacity. Neuroinflammation associated with COVID-19 may be involved in subsequent neurodegeneration. Alternative mechanisms by which this virus may putatively generate long term neuronal alterations could be related to an autoimmune response against α-synuclein, which seems to have a role in immune regulation and protection against viral infections. Taking into account all this information, we believe that there is a potential relation between SARS-CoV-2 and the pathogenesis of PD. Thus, a high degree of vigilance should be kept for the hypothetical role of this virus in neurodegenerative processes in recovered COVID-19 patients.

## Author Contributions

The authors declare contributed conception of the review. PC-C and PS-G wrote the first draft of the manuscript. All authors contributed to manuscript revision, read, and approved the submitted version.

## Conflict of Interest

The authors declare that the research was conducted in the absence of any commercial or financial relationships that could be construed as a potential conflict of interest.

## Footnotes

^1^Available online at: https://covid19.who.int
